# Supplementary material for: General population screening for type 1 diabetes using islet autoantibodies at the preschool vaccination visit: a proof-of-concept study (the T1Early study)
Source: Arch Dis Child. 2024 Jun 26;109(10):e326697. doi: 10.1136/archdischild-2023-326697 (PMC11503060; doi:10.1136/archdischild-2023-326697)
Supplement: online supplemental file 1 [file archdischild-109-10-s001.pdf]

Supplemental Materials

Supplemental Methods

Supplemental Methods 1: Participant information sheet.

Supplemental Methods 2. Blood collection kits

Supplemental Tables

Supplemental Table 1. Eligibility Criteria

Supplemental Table 2. Interview schedule

Supplemental Table 3. Questions asked on Postcards (2A) to participants, and (2B) to non-participants.

Supplemental Results

Supplemental Table 4. Self-reported ethnicity and family history of T1D collected using postcard questionnaires

Supplemental Table 5. Participant quotations from the data against the named themes and subthemes

Supplemental Methods

Supplemental Methods 1. Participation information sheet

Supplemental Methods 2. Blood collection kits

Sarstedt safety lancet 1.5mm blade, 1.6mm penetration and Sarstedt Microvette® serum gel capillary tubes with clotting activator (Sarstedt Inc., Newton, NC, USA).

Supplemental Tables

Supplemental Table 1. Eligibility Criteria

**Inclusion Criteria**
Parent/carer who is willing and able to give informed consent for participation in the study
A well child scheduled to attend a pre-school booster vaccination
Aged ≤ 5 years
**Exclusion Criteria**
Stage 3 T1D
Known coagulopathy or bleeding disorder
Parent/carer has insufficient understanding of written and verbal English

Supplemental Table 2. Interview Schedule

**Initial questions**	
**1.**	I wonder if you can tell me about your children, their ages, and the age of the child who took part in the recent vaccination/screening programme?
**2.**	Please can you now talk me through generally your experience of taking children to be vaccinated? (***Probe as necessary, what made you feel like that? Why?)***<br><br>a. Was this the first vaccination experience for this child or had they had previous vaccinations as a baby?<br><br>b. What are you general opinions on vaccination/screening?<br><br>c. Did your child/children have the heel prick test as baby? What did you think about this at the time?<br><br>d. What is your child generally like when you visit the GP/primary care surgery?<br><br>e. What were they like with previous vaccinations/injections for anything?<br><br>f. Have they been previously unwell which has required them to have a stay in hospital?

**3.**	You may remember that the study you took part in was to screen for the antibodies that appear in our blood and show if we are likely to develop Type 1 diabetes.<br><br>a. Did you have any knowledge of T1D before taking part in the study?<br><br>b. Do you have any family members with T1D?<br><br>c. Did you have any previous experience of diabetes?<br><br>d. What did you think about T1D? Why?
**Prior to vaccination/screening**	
**4.**	So I now want you to try and remember what happened when you were offered the vaccination/screening for your child.<br><br>a. Can you remember who contacted you?<br><br>b. What information were you given? How did you feel?<br><br>c. Were you given the opportunity to discuss it with other people?<br><br>i)Who did you discuss it with? Family member? Neighbour? Friend?<br><br>d. Did you look for further information yourself, independently?<br><br>i)Where did you look? What did you find out? Did this influence your decision to take part at all? *(Probe if they needed further information to take part, what sort of things did they want to know?)*
**5.**	Overall, what did you think about the opportunity to have your child screened for T1D?<br><br>a. Had you heard about T1D prior to the vaccination invitation? What did you understand about the condition prior to the vaccination day?<br><br>b. Were you worried or anxious about having your child screened? Why?<br><br>c. Did you have any other concerns? What were they?
**6.**	Overall why did you decide to have your child screened?
**Day of screening**	
**7.**	Please could you talk me through exactly what happened, step by step, on the day of the vaccination/screening? ***(Remember any probing questions...How did that make you feel? Why?)***<br><br>a. Was it you who took the child?<br><br>b. How was your child, happy? Worried? Upset?<br><br>c. Who administered the test/vaccine?

	d. Did your child have the screening (finger prick blood taken on the day)?<br><br>e. Or did you take a kit to do the test at home?<br><br>f. How did you feel the vaccination/screening went? Why?<br><br>g. What about the person delivering the vaccine/screening? How were they? What did you feel at the time?<br><br>h. What could have been done differently?<br><br>i. How was your child afterwards? If upset, how long before they were back to their normal selves?<br><br>j. What do you think the benefits might be for having your child screened?<br><br>k. Have you discussed the screening test with others? Who? Has this changed your opinion in any way?
**Whilst waiting for the results**	
**8.**	Have you had the results of the screening test yet?<br><br>a. Do you know when they are likely to arrive? And how they will arrive?
**9.**	Can you describe how you have felt whilst waiting for the results?<br><br>a. Have you been worried or not worried at all? If worried, what would have helped reassure you, if anything? Why? Have you sought reassurance from a health professional?<br><br>b. Have you sought further information, independently e.g. Google?<br><br>c. Overall would you describe the experience as positive or negative? Why?<br><br>d. Would you recommend it to other parents? Why?
**10.**	What would you think if the vaccination/screening programme was introduced throughout the UK?
**11.**	Would you have liked any other information?
**12.**	What could have been done differently?
**13.**	If made available, say as part of the schools' vaccination programme, would you have your child screened again? Say when they were 12 or 13? Why?

Supplemental Table 3. Questions asked on Postcards (2A) to participants, and (2B) to non-participants.

	**2A - Participants**	**2B – Non-participants**
**1.**	Tell us about your experience of taking part in T1 Early	Could you tell us why you decided not to take part in the T1 Early Study?
**2.**	How did your child find the finger prick blood test?	Is there anything else you would have liked to have known about the study, so that we can improve this in the future?
**3.**	Did you complete this card:<br>- At your child's vaccination visit?<br>- After your child's vaccination visit?	What were the advantages/disadvantages of receiving information electronically
**4.**	Why did you decide to take part in the T1 EARLY study?	What is your ethnic group?
**5.**	Is there anything else you would have liked to have known before taking part, so that we can improve this in the future?	Does anyone in your immediate family have type 1 diabetes? If yes, who?
**6.**	What were the advantages/disadvantages of receiving information electronically/doing remote consent?	

Supplemental Results

Supplemental Table 4: Self-reported ethnicity and family history of T1D collected using postcard questionnaires

	**Participants n=29**	**Non-participants n=3**
**Ethnicity**		
English/Welsh/Scottish/Northern Irish/British	22	2
Any other White background, please describe	1	0
Any other Mixed/Multiple ethnic background, please describe	1	0
Indian	1	0
Chinese	1	0
Not stated	0	1
**Family history of T1D**	3*	0

*1 unknown

Supplemental Table 5: Participant quotations from the data against the named themes and subthemes*

Theme	Sub theme	Quote (Interview or postcard)
Prior to the vaccination/screening visit	Opinion on vaccination and screening	*'I think it's really important and I think for sort of a couple of seconds of discomfort for her having it done, the benefits just outweigh…all of that peace of mind knowing that she's protected against anything.' (T1-31, Interview)*
Reasons for participating	Being prepared	*"I'd much prefer to just be informed of stuff. And if there's something I need to prepare for, I'd much prefer to prepare for it in a good amount of time, rather than suddenly finding out she's not well and panicking because she's not well, and then having to figure out how to live with it going forward. I'd much rather just have a plan." (T1-65, Interview)*
		"If he has got anything, we'll be safer knowing, and we'll be able to do all the right things." (T1-20 recalling her husband's response, Interview)
		'Because it was only giving a sample of blood and also if my child has type 1 diabetes it would be good to know about it and have the correct tools to deal with it.' (T1-48, Postcard)
	Ruling something out and feeling reassured	*'I just thought, why not get her tested, um, it's just another thing to make sure I don't have any concerns* (about). *But no, in terms of diabetes. Like, actually, her having diabetes, I wasn't actually concerned. No, I mean, it's just still reassuring because I don't know to what extent it can just, um, it's not genetic, so it could just pop up in a person when there's no family history, so I just thought, well I don't know that so there could be a risk.' (T1-39, Interview)*
		'I think, I mean, partly that knowing whether she had the antibodies it's, it's good to know, it's good to rule it out.' (T1-49, Interview)
		'Because I kind of find as a parent, …. like trying to have all these different appointments sometimes, you just

	Linked to vaccination programme	*can't do it. That is having it altogether is, just makes it so much easier.' (T1-13, Interview)*
		'I probably wouldn't have done it if it wasn't so easy. The fact it was being done at the same time, I didn't have to make another appointment.' (T1-3, Interview)
Reflections of the whole process	Child's response	*'He didn't even cry. But I said like this, this is amazing. He just did a bit of a bottom lip going, but then was fine. And that was just he loves stickers. So, he's very excited about the sticker he got and the plaster around his finger was the superhero or something. So, he couldn't wait to show his big sister. Ohh, went down a treat. Yeah. He loves that.' (T1-3, Interview)*
		'When I left, she was hysterical. I had to carry her out and like she was screaming.' (T1-19)
		'I mean, she was raving about it afterwards. All that evening, like, oh, look at my stickers that I got, you know, and I got the ice cream. So, she, I think she was very proud of herself.' (T1-39, Interview)
	Blood collection	*"So, I don't know how much blood is required, but I think that kind of bothered me a bit because she was sitting there, and the nurses had to really squeeze her finger quite hard to get the blood out." (T1-49, Interview)*
		'The only thing that struck me a bit, like maybe I've been naive with their kind of more squeezing the finger to get a decent amount of blood out.' (T1-20, Interview)
		'It was awkward because they had to push hard to get enough blood for the vial.' (T1-5, postcard)
		'I was not aware of the amount of blood that would be extracted. I was under the impression that the prick test would mean only a drop or two drops of blood. It would have been useful to have this information.' (T1-48, postcard)
		'He didn't mind the prick blood test, it was more the restraints he didn't like'. (T1-30, postcard)
		'Not too bothered - jabs were worse, so finger prick seemed lesser for her.' (T1-51, postcard)
		'Fine. Slight discomfort after. No tears.' (T1-61, postcard)

	Waiting for results	*'So, it hasn't been something that I've been like alarmed about. It's not like I've been part of the study and now I'm worrying about having diabetes.' (T1-67, interview)*
		'I've actually forgotten about it until this. (T1-29, interview)'
		'I'm sure some people could end up being a lot more anxious about it, if they worry about that kind of thing, but I'm not. Until it happens, I tend not to worry about it.' (T1-3, interview)
		'It's just popped into my head after. Probably after a month, you know, it's been a while, I think now. So, it just popped into my head. But no, it's not been like a constant worry. And she does like to drink. Sometimes drink quite a lot. So, I was thinking, ohh, she's drinking a lot, oh, better check. We've got this diabetes check, but actually I think she's fine.' (T1-39, interview)
	Benefits outweighing short-term upset	*'So yeah, for me, definitely, definitely positive. And I, I would do it again.'(T1-19)*
		'So I spoke to friends about it and explained it because I had a friend who had also been, their child had been invited to take part in the study. But she wasn't sure. She wasn't sure about it, and I just told her that, you know, it's, compared to their vaccinations, it's not anything, really.' (T1-22)
		'…that it took, it really took a long time because she kept saying to me ohh I'm nearly done, I'm nearly done, but she said that a few times, so all I remember thinking was like Jesus Christ, like he's gone through enough, hurry up.' (T1-63)
		'And yeah, and then they did the finger prick. I'm not sure whether they had to do it twice because of the, they had to like get more blood out, which was a bit sad on his behalf.' (T1-41)
Reasons for declining		*'Child upset by injections anyway. He will develop a fear of doctors and nurses if procedure is traumatic.' (002, postcard)*
		'Pre-school imms traumatic enough. Don't want to put him off doctors forever.' (003, postcard)

** Participant IDs have been anonymised*
